# A narrative review of pedagogical approaches and action pathways for climate change education in medical and health professions training

**DOI:** 10.3389/fpubh.2026.1769017

**Published:** 2026-03-25

**Authors:** Basima Maisoon

**Affiliations:** Independent Researcher, Sharjah, United Arab Emirates

**Keywords:** climate change, climate education, curriculum development, health education, medical education, sustainable development goals, public health education

## Abstract

**Background:**

The direct and indirect health consequences of climate change are increasingly becoming evident. To address the critical public health challenges posed by climate change, medical and health professions students need to be equipped with relevant skills, and institutions are adopting initiatives to integrate climate education. This narrative review aimed to explore implemented interventions for incorporating climate change and health education into medical and health professions training.

**Methods:**

Peer reviewed publications describing climate health interventions, were identified through database and supplementary searches. Data was synthesized narratively, focusing on pedagogical approaches, delivery and innovations.

**Results:**

Forty-one publications between 2020 and 2025 were reviewed. Six major themes emerged: integration into core curricula, flexible entry points (electives, workshops, and conferences), experiential and field based learning, advocacy and leadership training, faculty capacity building, and scalable and transferable models. Climate change and health education is being delivered in multiple formats, often combining didactic teaching with experiential activities. Scalable and transferable educational resources, learning models, and curricular integration strategies were identified. Student-led advocacy, institutional support and partnerships were drivers, while challenges include time, expertise and resource constraints.

**Conclusions:**

This review reflects gaining momentum in climate change and health education across medical and health professions training, particularly in recent years. The identified themes provide potential action pathways for integrating climate-health interventions.

## 1 Introduction

**Figure 1:**
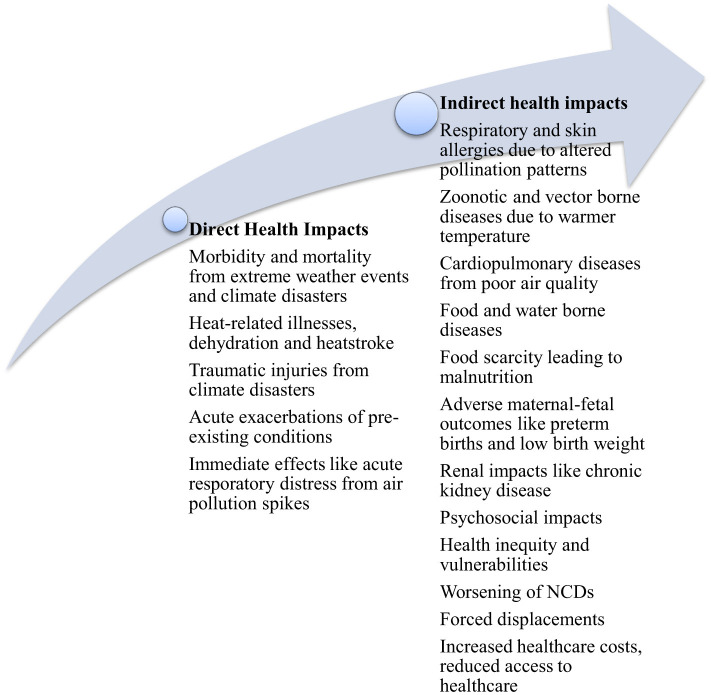
Direct and indirect health impacts of climate change.

The current climate change (CC), which is predominantly anthropogenic in origin, poses significant public health threats and is projected to cause additional 250,000 deaths per year between 2030 and 2050 (1). Climate events such as heat waves, droughts, poor air quality and other extreme weather conditions and climate related disasters cause several direct and indirect health consequences (1), as shown in Figure 1. These have profound implications on health systems worldwide. Considering the escalating and critical impacts of CC on public health, it becomes imperative for medical and health professionals to be equipped with the knowledge and skills to understand and mitigate them.

The role of medical and health professionals in addressing CC is multifaceted and interdisciplinary, including clinical, educational, public health and policy areas. Clinicians and other health professionals treat and manage climate related health issues across diverse settings. In addition, they may have to identify health risks and vulnerabilities associated with CC, at both individual and population level, engage in public health communication, and educate patients and communities on climate related threats. They may also have roles in imparting medical and health education by teaching students, developing curricula, and integrating climate considerations into training programs. They are also well-positioned to support resilient health systems and promote sustainable healthcare practices. Many professionals also engage in leadership and advocacy, and can contribute perspectives to inform policy decisions related to CC adaptation and mitigation in the health sector.

In recent years, there has been growing consensus on the importance of imparting climate change and health (CCH) education for students of medicine and other health professions. Climate-health experts, student organizations, professional bodies, and education councils have emphasized this need (2, 3). In response, several institutions and programs have begun incorporating CC education, though the extent and instructional methods vary across settings (2, 4).

Considering this evolving scenario in medical and health professions education, this narrative review was undertaken to synthesize recent evidence on how CCH education is delivered globally. This would offer insights for educators and curriculum developers, contributing to advancing SDGs 3 (good health and wellbeing), 4 (quality education) and 13 (climate action). An exhaustive coverage of every institution is not attempted; instead, the focus is on pedagogical approaches including learner engagement models, instructional strategies- both theoretical and experiential, and innovative approaches adopted worldwide.

Accordingly, the objective of this review is to explore and synthesize contemporary pedagogical approaches and action pathways used in CC education in medical and health professions training by analyzing the body of evidence published in recent years documenting such initiatives.

## 2 Methods

This review was conducted as a narrative synthesis of recent pedagogical approaches to CCH education in medical and health professions training. The review process and reporting were guided by SANRA (Scale for the Assessment of Narrative Review Articles) to ensure overall rigor (5).

### 2.1 Search strategy

A literature search was conducted in PubMed, using the following the search string:

[(climate OR “climate change”) AND (“medical education” OR “medical curriculum” OR “health professions education”)].

In addition, a topic guided internet search was conducted to identify supplementary relevant materials.

The final search was performed on 13 December 2025.

### 2.2 Study selection criteria

Peer-reviewed articles that describe teaching or inclusion of CCH topics within medical and health professions education or training, with publication year 2020–2025, were included.

Articles focusing solely on student or stakeholder readiness, perspectives, needs assessment, and state of climate education, without describing curricular content or pedagogical strategies were excluded. Studies that noted the inclusion of CC topics but did not provide details on instructional methods, content, or implementation were also excluded.

### 2.3 Operational definitions

In this review, the term “intervention” is used broadly to denote any structured educational activity aimed at delivering CCH education within medical and health professions training. The term “medical and health professions” refers to professions involved in providing healthcare and treatment for humans, based on formal professional education and training.

## 3 Results and analysis

Publications on CCH education in medical and health professions training were identified through the searches. Articles providing sufficient details on curricular content, teaching strategies, and implementation were included in this review, resulting in 41 studies for analysis. These studies are summarized and synthesized below.

### 3.1 Characteristics of included articles

All the included articles described a CCH intervention such as an elective, module, workshop, seminar or other instances of learning or curricular integration; or outlined novel curricular development and implementation, with date of publication ranging from 2020 to 2025. The article types ranged from purely descriptive ones like scholarly perspectives, commentaries, curriculum and pedagogy reports, to primary research studies employing quantitative, qualitative or mixed methods designs. The research studies mostly consisted of quantitative designs which used pre and post intervention surveys to assess learners' knowledge and perceptions before and after the CCH intervention. Qualitative designs like focus group discussions, analysis of reflective essays, progress reports and other open ended feedback analysis were also found.

The selected articles represent diverse geographical distribution. Most of the included studies originated from the United States (*n* = 24), followed by Germany (*n* = 6), the United Kingdom (*n* = 2), and Canada (*n* = 2). Single studies were identified from Switzerland, France, Norway, Austria, and Australia. In addition, two initiatives described multinational reach, including a multi country African alliance.

### 3.2 CCH interventions and target groups

The review identified a wide range of CCH educational initiatives aimed at different stages of medical and health professions training. Table 1 shows an overview of these interventions and the target groups. Interventions span undergraduate, postgraduate medical and health professions students, as well as climate fellowships.

**Table 1:** Overview of CCH interventions by target groups (*n* = 41).

**Category**	**Target groups**
Curricular integration and other mandatory formats in undergraduate medical education	• Preclinical stage (7, 9, 12, 19, 23, 26, 29, 36) • Clinical stage (11, 34, 35) • All years (6, 10, 16) • Pre-medical students (22)
Curricular integration and other mandatory formats in postgraduate medical training	• Pediatric residents (13, 28, 30) • Internal medicine residents (30) • Family medicine, and social medicine residents (31) • Family medicine, OBG residents (32) • All postgraduate training (49) • GP residents (33) • Junior doctors (6)
Electives and optional courses, modules	• Undergraduate medical students (8, 16, 17, 20, 21, 27, 37, 38) • Graduate and undergraduate medical and health professions students at all schools of a university (25) • Graduate health professions students of two universities including occupational therapy, physical therapy, nursing, and emergency and disaster Medicine, speech language pathology (39) • Audio podcast for first year nursing and mid wifery students in an institution (15)
Fellowships	• Graduate medical education fellowship for physicians (44, 45) • Fellowship for medical and health professionals including physicians, nurses, and medical students (18)
Open digital interventions with national or global reach	• Post licensure medical and health professions from 25 countries (43) • UG medical students across 13 Canadian medical schools (29) • Medical and health professions students and professionals across Germany (42) • Medical and health professions students globally (14)
Workshops and conferences	• Workshop with participants from 40 countries in Africa, included medical students, residents, medical officers, nurses, researchers (40) • Workshops for undergraduate medical students in three campuses (24) • Conference for pre-health students, medical and health professions students- including medical, nursing, physician assistant, dental, allied health, health research students (41)

Some studies are represented in more than one category as they targeted multiple learner groups or combined different interventional formats.

### 3.3 Drivers of CCH interventions

**Figure 2:**
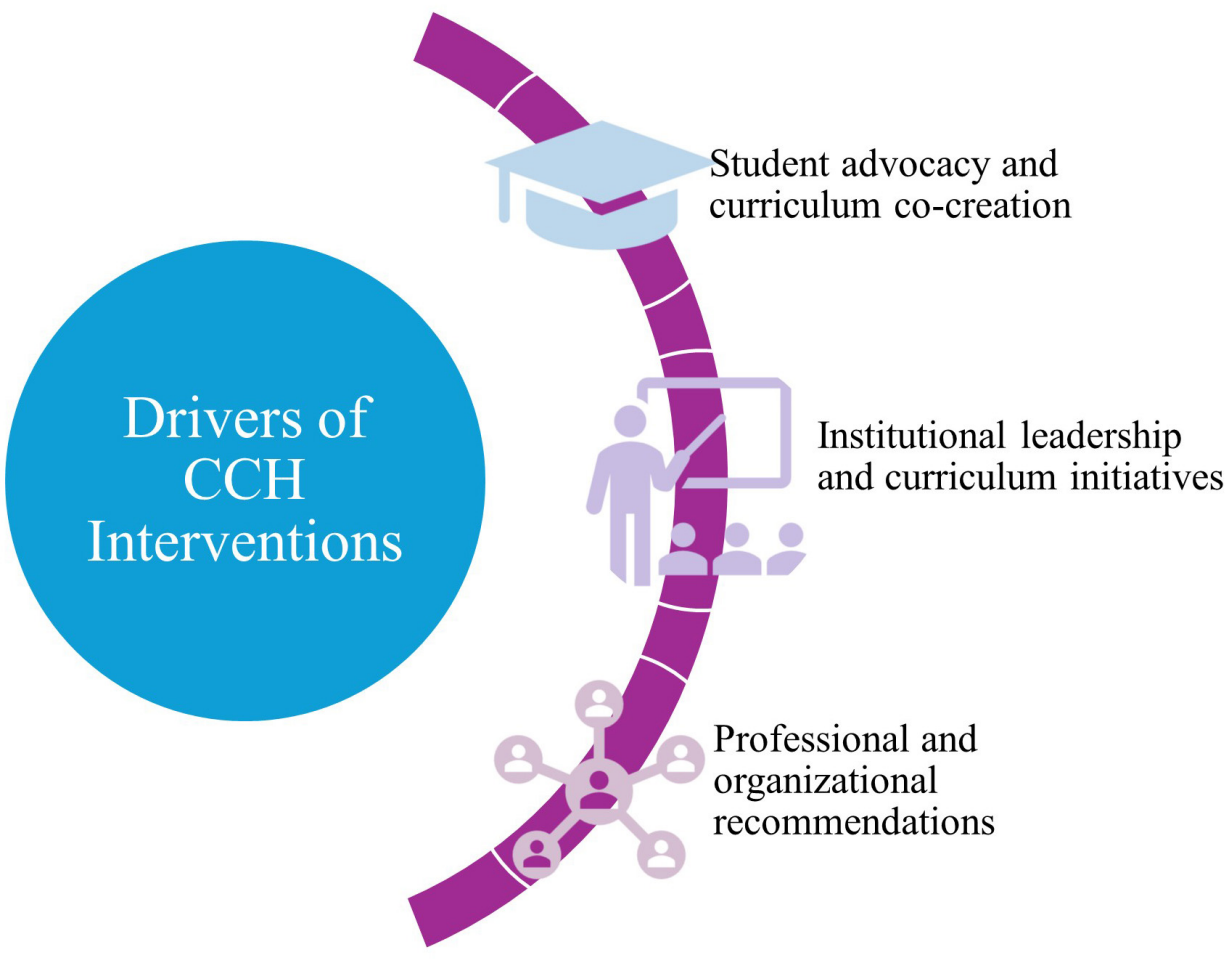
Drivers of CCH interventions in medical and health professions education.

Several factors were identified as drivers of CCH interventions. These include student, institutional and professional levels, as depicted in Figure 2. Student-led advocacy was a prominent motivator, with students actively promoting CCH curricular initiatives (6–11). In some instances, students co created curriculum and developed CCH content with faculty (7, 11–17). Institutional leadership motivated and played key role in several cases (11, 18–27). Recommendations from medical education councils, initiatives by climate focused groups, and other alliances were also important drivers. For example, a pediatric residency program incorporated a session on CCH in its first-year curriculum, responding to American Medical Association's call for climate education (28). Collectively, these findings highlight multilevel efforts in shaping and materializing CCH interventions.

### 3.4 Content of CCH interventions

**Figure 3:**
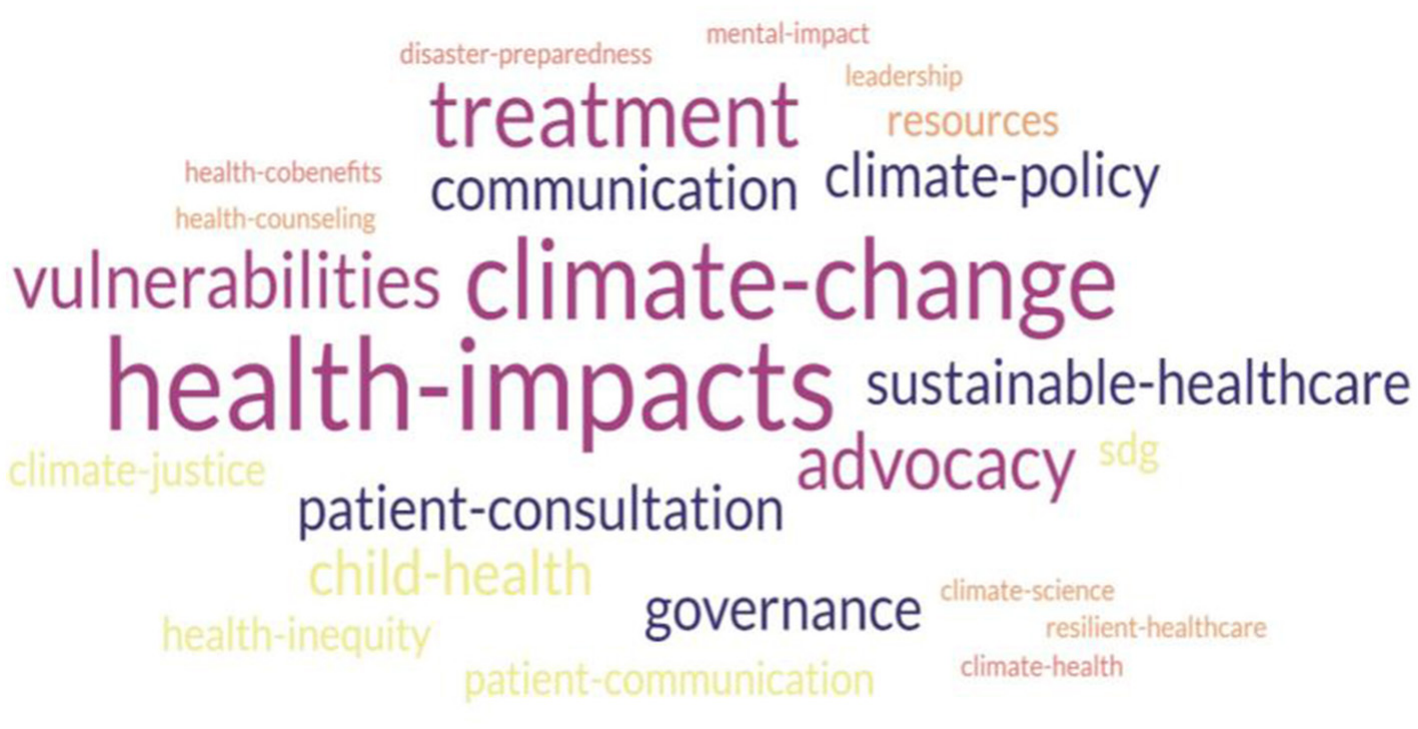
Content analysis of theory and didactics.

Content analysis of the CCH interventions identified two broad categories—theoretical and practical learning. Most interventions included theoretical components on foundational topics such as climate science, planetary health, environmental determinants of health, impact on health and health systems, principles of management of health impacts, principles of sustainability, vulnerabilities, climate justice, health inequities, Sustainable Development Goals, health co-benefits of climate action, resources available for CCH education etc. Reported climate related exposures include heat events, air pollution, environmental contamination such as heavy metal and toxin exposure, and climate sensitive hazards causing food and water insecurity. Associated health effects include respiratory illnesses, allergies, vector borne and water borne diseases, malnutrition and childhood nutrition challenges, mental health impacts, trauma, maternal and fetal health impacts, and cardiovascular morbidity. These themes were generally delivered across all learner groups. Targeted and specific content such as mental health impacts of CC in psychiatry, and climate-related risks and management in pediatrics were also identified. The key themes in theory are visually summarized as a word cloud in Figure 3 content analysis of theory and didactics.

**Figure 4:**
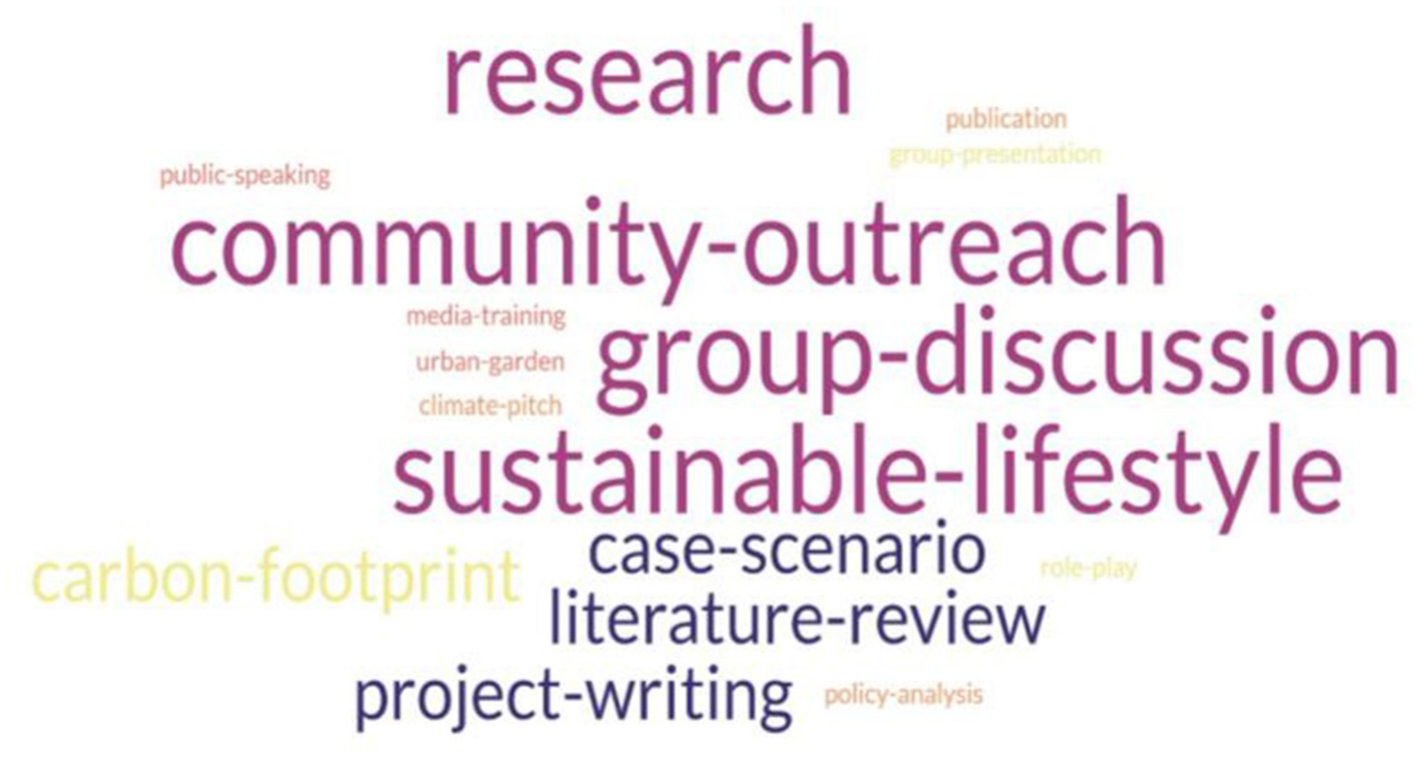
Content analysis of practicum.

In addition to traditional lectures, a wide range of practical learning and delivery formats were used to facilitate learning. These included small group discussions, case studies, journal club presentations, writing reflection papers, role plays, climate pitches, calculating ecological footprint, adopting sustainable lifestyles, using assessment tools, clinical management of climate-related health impacts, interprofessional collaboration, media and advocacy training, policy and governance training, producing publications, and conducting research. Many interventions included community outreach programs. The key themes in practicum are shown as a word cloud in Figure 4 content analysis of practicum.

### 3.5 Thematic analysis of pedagogical approaches

The following themes emerged from the included studies, based on their educational purpose. These themes illustrate action pathways through which CCH can be included in medical and health professions training. Many interventions combined multiple educational strategies and delivery formats.

#### 3.5.1 Theme 1: integration into core curriculum

This theme includes interventions that integrated CCH into the core curricula and existing study plans (6, 7, 9, 12, 13, 16, 18, 19, 22, 23, 26, 28–32). Such integration reflects inclusion of CCH as a standard and routine component of education, rather than as an optional or supplementary topic.

Among these, some interventions adopted longitudinal integration through multiple years of training (13, 16, 30), supporting progressive learning. Other instances include incorporation of CCH as part of a new and extensive curriculum module on environmental health (23). In several instances, CCH components were embedded into parts of existing courses or modules (12, 22, 26, 28, 31, 33).

Importantly, some studies demonstrate that short and focused instructional units and modules can also be used to effectively integrate CCH into core training and curricula (33–36). These ranged from 45 to 60 min sessions to brief blended learning modules that were incorporated into undergraduate training, clerkships, and residency programs.

These interventions show that institutions can successfully integrate CCH into core curriculum by several pathways including modifying longitudinal curricula, introducing focused mandatory modules, embedding content within existing courses and through short well-designed instructional units that fit into existing clinical training. This underscores that to impart meaningful CCH education, extensive curricular redesigning is not always necessary, rather strategic use of existing structures can be both effective and feasible. Importantly, integrating into core curriculum ensures that all the learners are formally exposed to CCH as a core competency.

#### 3.5.2 Theme 2: flexible entry points

Flexible entry points include elective courses, conferences, and workshops that allow institutions to introduce CCH content without immediate large scale curricular reforms. Several institutions offered successful electives on CCH (8, 17, 20, 21, 25, 27, 37, 38). For example, a mandatory elective “climate change and health” was offered as a 28 h block seminar, providing comprehensive coverage of the scientific foundations, health impacts, mitigation strategies etc. (20). Other formats included a 2.5 week long symposium, which included lectures and discussion with CC experts, and case studies (39), workshops (24) and conferences (40, 41) which provided focused learning.

Digital and online interventions also offered flexible curricular use like self-directed modules, open lecture series, and short learning resources which could be used readily by integrating into curriculum or accessed independently by learners (14, 37, 42). In one model, along with longitudinal curricular integration in undergraduate years, electives were also offered, thereby creating opportunities for interested students to develop additional knowledge and skills (16).

Flexible approaches are relatively easy to implement and provide focused and time bound learning of CCH. They are particularly useful when immediate curricular reforms are not feasible- allowing institutions to pilot content and generate faculty and student interest. At the same time, their non mandatory nature might mean that students who are already motivated or climate conscious enroll in these courses more than others. Nevertheless, they can be adopted as important first step toward CCH integration, or as complementary opportunities to gain deeper knowledge.

#### 3.5.3 Theme 3: experiential and field-based learning

This theme includes experiential learning approaches identified in the studies, which emphasize engagement with real world clinical and community contexts. Several interventions used case studies, simulations and role play to build climate health competencies, including diagnosis, counseling, patient communication, and disaster preparedness (16, 28, 31, 35, 43). Analysis of clinical scenarios, information synthesis from multiple sources, group projects and presentations were also commonly adopted (35, 38, 39). Students conducted literature reviews to analyze and summarize scientific publications on CCH and wrote projects (20, 25). Other activities included evaluating current political measures regarding CC and interventions focusing on developing actionable skills in clinical, administrative, and research domains (20, 34).

Community based learning activities such as community organizing, project development, and engagement with local organizations helped to situate learning within real social contexts (12, 16, 18, 25). Other experiential components like using assessment tools to analyze own diet and personal care choices, writing reflection papers, and calculation of individual ecological footprint were helpful to connect professional responsibilities with personal behavior (21, 23, 26).

Fellowship programs represented an advanced form of experiential learning. These included professional outputs like peer-reviewed journal publications, professional and academic talks, poster presentations and grants, as well as engaging in teaching activities for trainees, peers, and the public (18, 44, 45).

This theme shows that experiential learning can be incorporated at all levels of training and education. Institutions can strengthen CCH education by prioritizing experiential components, even through small scale activities. Such approaches improve practical competence and the learners' capacity to translate knowledge into action.

#### 3.5.4 Theme 4: advocacy and leadership training

This theme highlights efforts at advocacy and leadership training in climate and health. Examples are a climate policy and advocacy workshop (24), and a “climate justice and health equity” curriculum in the advocacy training of a pediatric residency (13). Training in communication, governance, and leadership was also emphasized across several interventions (6, 24, 31, 44, 45). Some interventions encouraged broader public engagement like writing op-eds in newspapers (17). Fellowship programs were found to strengthen this dimension by including media and advocacy training, policy development, governance, and community engagement (18, 44, 45).

These interventions demonstrate that advocacy and leadership training can connect professional roles with social and environmental accountability. By preparing learners for advocacy and leadership and become agents of change, such interventions address a gap in traditional medical and health education. Climate advocacy fits with professionalism and ethics, and is closely related to health equity and justice, hence such approaches merit wider adoption.

#### 3.5.5 Theme 5: faculty capacity building

This theme emphasizes the role of faculty and capacity building as central for sustainable CCH education. Some studies show that a barrier to implementation of CCH interventions is the limited number of educators who are confident in teaching CCH (29, 34). To overcome this, the development of structured, ready to use materials such as slide sets, and facilitator guides were shown to be efficient. These resources enabled impactful CCH interventions, even when prior faculty expertise was limited (29, 34, 39).

Faculty co learning was an effective strategy. In one model faculty and students were enrolled as learners and taught by climate experts, while simultaneously contributing to course development (39). This approach showed that collaborative expert input can contribute to curriculum design even by climate novice educators. In another instance, curriculum was co-created with regional environmental health experts (31). Use of existing open access materials were also mentioned as effective for capacity building among faculty (13). Partnerships to implement the interventions can be instrumental, as well as motivation and readiness from the faculty. In this regard identification of well-motivated climate champions is also important (19, 39). A student driven collaborative model supported by faculty and administrators highlighted the role of stakeholder involvement in capacity building and curriculum development (16).

These studies highlight that faculty capacity building for CCH interventions is both feasible and achievable. With resources and support, even educators without prior expertise can develop and deliver CCH content. Moreover, motivated educators combined with strong institutional ownership can ensure the sustainability of interventions.

#### 3.5.6 Theme 6: scalable and transferable models

Among the included studies, several interventions had potential for scalability with many authors explicitly recommending their uptake or adaptation by other institutions (11, 13, 16, 29, 36–38, 42, 43).

A notable feature is the creation of open access, and shareable educational resources. Examples include the “climate wise slides,” an evidence based open access didactic tool, developed in Canada, and intended for use by medical education systems globally (29). Similarly, the “planetary health academy,” the first open online lecture series for climate education in Germany provides guidance for transformative education approaches (42). The “climate change and human health ECHO (CCHH ECHO),” tele-mentoring series by a peer learning network, also demonstrated global uptake (43).

Other resources included an online module “climate change and sustainability in clinical practice” for clinical phase medical students (37), and a set of short online learning resources “bricks” on planetary health, among which several were on CCH, developed on a digital education platform by student educator collaboration from multiple countries (14). Some of these interventions had global reach (14, 42). These accessible and shareable resources provide scalable entry points for integration of CCH in different contexts.

Several interventions demonstrated innovation that enabled integration without extensive curricular changes. The “climate change curriculum infusion project (CCCIP)” allowed extensive integration of CCH while minimally disrupting existing course work, by strategically identifying insertion points within current curriculum (9). Similarly, curricular developments like a “climate change, health, and equity” curriculum thread (12), a “climate justice and health equity curriculum” within the advocacy training of a pediatric residency program (13), and a longitudinal pediatric residency curriculum including CCH (30) show unique models across specialties and training levels.

The “Climate-LIMETTE” initiative in Germany evolved into a mandatory component of undergraduate medical curricula, including OSCE (Objective Structured Clinical Evaluation)-based simulations with simulated patients, and formative assessment (11). This model highlights how assessments can anchor CCH education within existing training structures.

Similarly, the “Planetary Health Task Force” functioning in a U.S institution is an example of a comprehensive bottom-up intervention, with initiatives such as longitudinal curricular integration along with implementation of electives, core competencies development, mandatory waste management training, environmental exposure screening and counseling, student-led research, and community engagement (16).

Climate policy and advocacy workshops were also noteworthy as the participants were able to build skills related to communication on climate policies, and engage in advocacy, thus addressing the gaps in CCH related advocacy within core curricula (24).

### 3.6 Assessments and evaluation

Many studies reported post intervention assessments to evaluate participants knowledge, skills, attitudes, and similar outcomes related to CCH after the intervention (12, 14, 17, 21–23, 32, 35, 41, 42). Pre and post interventions were also employed in many instances to assess changes attributable to the intervention (13, 15, 18, 28, 31, 33, 34, 36, 37, 44).

Assessment methods were predominantly quantitative, most commonly using survey questionnaires. However, qualitative evaluation methods like feedback analysis, progress report analysis, personal interviews, focus group discussions, open ended questions, graded essay writing, etc. were also reported capturing insights like learners' perceptions and recommendations on content relevance. Many studies adopted mixed methods designs. For example, a study conducted comprehensive evaluation using post intervention personal interviews of participants, course evaluation using structured forms, and pre and post intervention survey on student knowledge and awareness of CCH (20).

### 3.7 Barriers and enablers to implementation of CCH interventions

Some recurring challenges in implementation of CCH interventions were identified across the studies. These challenges were at institutional, curricular, resource related and student levels.

Institutional and curricular barriers included resistance to integrating non-traditional topics into established biomedical curricula, (7, 19), concerns about displacing existing content (7), limited curricular time, and scheduling constraints (7, 8, 19). To overcome these, broad stakeholder involvement and co creation of learning by students, administrative members and faculty were effective (7, 19). Embedding CCH content within existing modules were recommended in some settings (16, 23). Additionally, CCH content was designed to fit curricula, without significant schedule changes (8, 16).

Another challenge was the lack of availability of faculty with sufficient expertise and confidence to develop CCH curriculum or to teach climate-health content (34). Difficulties in recruiting climate health expert facilitators were also reported (13, 19). In a setting, faculty and students enrolled as co-learners in content delivered by climate experts. This enabled the faculty to improve their climate proficiencies and to develop further institution specific climate courses (39). Use of open-source teaching materials, facilitator guides and resources are recommended to support faculty to improve their expertise (13, 34). Beyond expertise, identification and recruitment of committed faculty climate champions was reported as important to the success and continuity of some CCH interventions (19, 39).

Resource constraints including funding were also noted especially in the case of fellowships. Institutions considering similar initiatives were recommended to check access to mentorships, external funding and additional nonclinical resources (44).

At the student level, resistance to nontraditional topics like CCH that were not relevant to medical licensing examinations, was noted (7). The inherent limitation of electives, being that those who enrolled might be previously climate conscious students compared to those who did not enroll, leading to loss of learning opportunity for the latter, was highlighted (20). Core curricular integration and long term programs were recommended as solution for the challenges posed by electives (8, 23). However, it was also noted that while integrating CCH to core curriculum is essential, starting with an elective may be a feasible and efficient way to bring about awareness and generate interest on the subject, among students and faculty (38).

While student led innovation were frequently described as key drivers of innovations, studies emphasized that sustainability of the interventions require institutional ownership, rather than rely on the voluntary commitment of students (8, 14). Achieving transformative learning outcomes and action-oriented change, beyond mere knowledge of CCH, was identified as challenges, though it was also reported that even online groups and lectures that impart practice oriented climate education might have the potential to motivate learners in those directions (42). Strategies like personalized coaching (in fellowships), simulation-based training, creative outputs, community organizing training, were reported to improve engagement and applied skills for climate action (18, 24, 27).

## 4 Discussion

This narrative review synthesized contemporary pedagogical approaches to CCH education across medical and health professions training worldwide through a thematic analysis of implemented interventions. Interventions varied in content, integration strategies, delivery modes, and evaluation methods, yet collectively demonstrated growing momentum in including climate related topics in health education. Evidence from 41 instances of CCH education informed this review.

Most interventions include didactic teaching covering the fundamentals and basic science of climate change, health impacts, systemic impacts, sustainability, vulnerabilities, health equity and justice, advocacy etc. Some programs delivered specialized content according to their focus area, like child health and mental health. Interactive, experiential and action oriented learning content and group activities were increasingly incorporated, reflecting competency development. Governance, leadership training, and community engagement were included in some settings, though less widely adopted than conventional and didactic approaches.

A notable feature of this review is the identification of transferable, adoptable and scalable models, including open access resources, teaching materials and curricular infusion strategies. These also provide insights for future implementation based on feedback and evaluation.

Further, this review highlights diverse opportunities to integrate CCH with respect to type, duration, scope, mode of delivery, and teaching approach. CCH content can be incorporated in multiple ways- including stand-alone modules, elective courses, longitudinal threads, embedded curricula, workshops, symposia, and open online resources. Key drivers and enablers include student involvement, faculty and institutional support, and partnerships. Notably, climate expertise of faculty was not always necessary for initiation and impact of an intervention. Stakeholder involvement, partnerships, and utilization of local resources also proved beneficial.

The review also shows a lack of standardization in CCH courses as well as curricular integration. Longitudinal integration and mandatory modules were found only in some settings. Though CCH electives were popular, their optional nature might have meant limited reach. Beyond knowledge acquisition, there are also gaps in transformative learning outcomes, calling for strategies that promote learners toward actions and engagement. These highlight the need for structured integration, faculty development, and institutionalization of interventions.

The findings of the current review extend earlier literature on the subject. Growing interest in climate health education has been documented previously (46, 47). A comprehensive 2022 review on planetary health in medical education found that while many publications proposed learning objectives, very few included implementation perspectives and information on full curricular development (46). A scoping review summarized students' competencies, conceptual frameworks and teaching methods related to planetary health broadly, providing significant conceptual overview (47). Another narrative review focused on the knowledge, attitudes, and needs assessment of students regarding CC education while noting teaching activities and identifying possible content (48). The current review complements existing literature, by focusing on implemented CCH educational interventions within medical and health professions education. It moves from rationale, frameworks and learner perceptions, toward how CCH has been delivered in practice between 2020 and 2025. By synthesizing pedagogical themes including delivery, implementation, and scalability considerations across medical and health professions education, the current paper provides practice-oriented insights into how CCH education can be effectively designed, implemented, and sustained. Though the current study did not aim to compare interventions by country using an exhaustive mapping of all initiatives, the findings are consistent with previous reviews in indicating that most climate education interventions originate from high income countries, with United States being most represented, and that publications are predominantly led by authors from Europe and North America (46, 47).

### 4.1 Limitations

This review did not include every published study on the topic, nor was a systematic search of gray literature conducted. As a result, some interventions may not be included. Additionally, it does not assess the relative effectiveness of interventions, rather it provides a descriptive synthesis and actionable insights for educators and institutions.

## 5 Conclusion

This review highlights the increasing integration of CCH within medical and health professionals training globally. Wide range of approaches are used including mandatory modules, elective courses, longitudinal curriculum integration, workshops and conferences. Most combine didactic and experiential learning and cover fundamentals of CCH, while some deliver focused content. The initiatives are frequently driven by student-led advocacy, institutional support, and strengthened through partnerships with climate organizations and alliances. The findings offer actionable insights for educators, institutions, and other stakeholders seeking to implement or scale climate education.

## Data Availability

The original contributions presented in the study are included in the article/supplementary material, further inquiries can be directed to the corresponding author.
